# From laboratory to clinic: a precise treatment strategy of mesenchymal stem cells-derived exosomes pretreated by simulating disease microenvironment

**DOI:** 10.3389/fimmu.2025.1609288

**Published:** 2025-07-17

**Authors:** Hailian Ye, Qi He, Fang Qi, Guangchao Xu, Mulan Qahar, Chenliang Deng, Zairong Wei

**Affiliations:** ^1^ Department of Burns and Plastic Surgery, Affiliated Hospital of Zunyi Medical University, Zunyi, Guizhou, China; ^2^ The 2011 Collaborative Innovation Center of Tissue Damage Repair and Regeneration Medicine, Affiliated Hospital of Zunyi Medical University, Zunyi, Guizhou, China; ^3^ The Collaborative Innovation Center of Tissue Damage Repair and Regeneration Medicine, Zunyi Medical University, Zunyi, Guizhou, China

**Keywords:** mesenchymal stem cells, exosomes, disease microenvironment, inflammation, hypoxia, tissue repair, precision medicine, miRNA delivery

## Abstract

Mesenchymal Stem Cells (MSCs) and their secreted extracellular vesicles (EVs), particularly exosomes (Exos), have garnered significant attention for their potential in tissue repair, fibrosis, and tumor therapy. However, the therapeutic efficacy of mesenchymal stem cell-derived exosomes (MSC-Exos) is notably influenced by the disease-specific microenvironment. This review examines the mechanisms of action of MSCs and MSC-Exos in various diseases and analyzes the impact of inflammatory preconditioning on the functions and paracrine signaling of MSCs. We propose a personalized MSC preconditioning strategy based on the characteristics of the disease microenvironment to enhance the precision and efficacy of MSC-Exos therapy. Additionally, we discuss the limitations of traditional preconditioning strategies and introduce novel approaches for MSC preconditioning by simulating the disease microenvironment, such as using tissue homogenates and EVs derived from diseased tissues. These methods more accurately reflect the spatiotemporal features of the disease microenvironment, thereby improving the therapeutic potential of MSC-Exos. Finally, we explore the application of engineered exosomes loaded with key miRNAs targeting disease treatment, offering new insights for precision medicine.

## 1 Introduction

In recent years, the rapid advancement of regenerative medicine and cell therapy technologies has propelled mesenchymal stem cells (MSCs) and their derivatives, particularly exosomes, to the forefront of novel therapeutic strategies (1–3). Studies have demonstrated that exosomes (MSCs-Exos) secreted by mesenchymal stem cells (MSCs) exhibit biological effects akin to those of MSCs (4–6). In the process of wound repair, MSCs-Exos can enhance the local microenvironment by modulating inflammatory responses, promoting angiogenesis, and facilitating cell proliferation and migration, ultimately contributing to improved wound healing (7–9). Compared with MSCs, MSC-Exos possess several advantages, including high efficiency, long-term stable storage, ease of transportation, and dose control, and almost no risk of tumor and thrombosis (10). These advantages make MSC-Exos regarded as an alternative treatment option to MSCs and have broad clinical application prospects (2, 11, 12). However, these biological agents exhibit significant heterogeneity in actual use. It is generally believed that cell source, culture environmental factors, and the local pathological microenvironment at the transplantation site affect cell growth, function, and the paracrine system (13–15).

**Figure 1:**
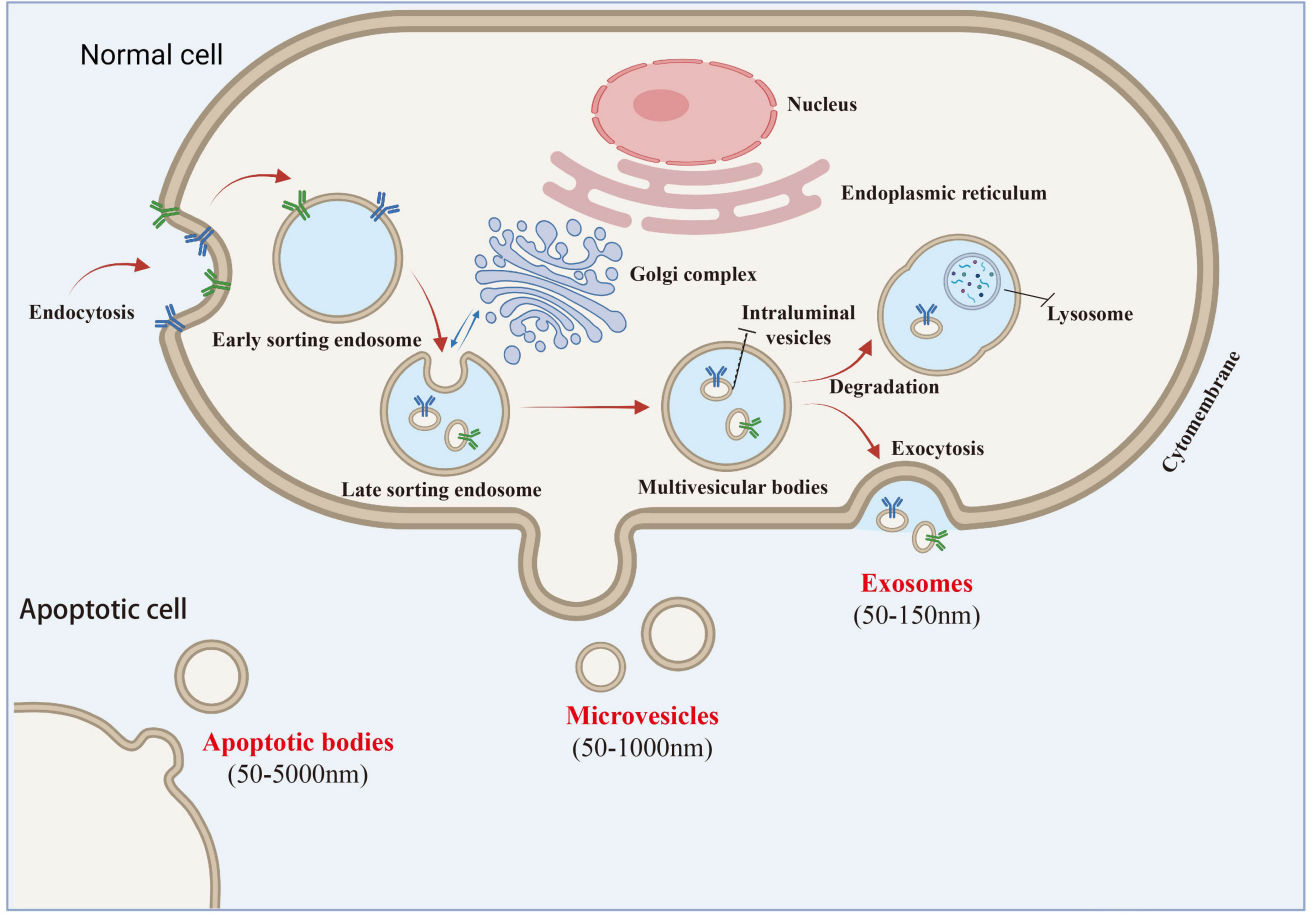
Extracellular vesicles and exosomes secretion pathway diagram.

Exosomes are a subtype of extracellular vesicles (EVs) with a lipid bilayer structure and sizes ranging from 30 to 150 nm. According to the 2014 guidelines on the “ Minimum experimental requirements for extracellular vesicles and their functions” (MISEV2014) established by the International Society for Extracellular Vesicles (ISEV), the term “exosome” has emerged as the most frequently used descriptor for “extracellular vesicles” in the literature since 2004. In contrast, “EVs,” introduced by the International Society for Extracellular Vesicles (ISEV) in 2011, refers to a heterogeneous group of particles released from cells through various pathways and involved in intercellular communication (16). The 2018 ISEV guidelines (MISEV2018) defined EVs as lipid bilayer-enclosed particles lacking a functional nucleus. EVs include exosomes, large vesicles, microvesicles, microparticles, and apoptotic bodies. However, specific markers for these subtypes remain elusive due to challenges in identifying subcellular origins. The guidelines recommend naming EV subtypes based on physical characteristics (e.g., size or density), specific surface markers (e.g., CD63+/CD81+), or conditions/cellular sources (e.g., hypoxic EVs) Despite ongoing nomenclature debates, the term “exosome” is still widely used (17). Despite the ongoing debate regarding nomenclature, the therapeutic potential of these subcellular components remains unchanged, and the term “exosome” continues to be widely adopted by researchers due to its familiarity. Notably, the 2023 guidelines on extracellular vesicles underscore the necessity for precise terminology and experimental rigor in EV research, emphasizing the importance of distinguishing between different EV subtypes based on their biophysical properties and biological functions. This precision is essential for advancing our understanding of EV biology and optimizing their therapeutic applications (18) (**Figure 1**).

MSC-Exos are capable of carrying a diverse array of biologically active molecules, including proteins, mRNA, and miRNA (19). They can not only transmit signal factors to regulate the growth, differentiation, and function of target cells but also serve as nano-carriers for targeted drug delivery, thus being widely applied in tissue repair, inflammation reduction, and fibrosis inhibition (20, 21). Although MSC-derived extracellular vesicles (MSCs-Exos) have demonstrated significant potential in basic research, their clinical application continues to encounter numerous challenges. Most current studies focus on culturing MSCs under traditional conditions to isolate extracellular vesicles (EVs) and subsequently investigate their therapeutic effects on various diseases (22). While notable efficacy has been observed, this approach may overlook the influence of the disease microenvironment on MSCs. A critical issue is that the biological effects of MSCs-Exos exhibit considerable variation across different disease microenvironments (13).

For instance, in chronic wounds, MSCs upregulate the expression of vascular endothelial growth factor (VEGF), thereby promoting angiogenesis and facilitating wound healing (23). Conversely, in malignant glioma, MSCs downregulate VEGF expression within tumor tissue, which serves to inhibit tumor growth (24). Even within the same disease context, adipose mesenchymal stem cells can enhance collagen I and III expression in fibroblasts when transplanted into open wounds, accelerating collagen synthesis and granulation tissue formation to promote wound healing (25). However, when these cells are transplanted post-epithelialization, their paracrine effects lead to a downregulation of collagen synthesis, counteracting fibrosis and scar formation (26). This discrepancy indicates that the therapeutic efficacy of MSCs-Exos is not solely reliant on their intrinsic biological properties but is also intricately linked to the ‘temporal’ and ‘spatial’ characteristics of the disease microenvironment (27). We have synthesized our thinking on this finding, current research on mesenchymal stem cell-derived exosomes (MSC-Exos) ignores an important scientific issue: the lack of targeted induction or preconditioning of MSCs for therapeutic use in disease pathological microenvironments. In particular, it is not fully considered that when mesenchymal stem cells are used as cell grafts, their paracrine composition and biological effects may change depending on the pathological microenvironment of the disease.

Consequently, to enhance therapeutic outcomes, researchers have begun to pre-treat MSCs cultured *in vitro* in accordance with specific disease microenvironmental features (such as inflammation and hypoxia) to modify the biological functions of the EVs secreted by MSCs and improve their therapeutic efficacy in related diseases (28). To further enhance the therapeutic effect, researchers have started to pretreat MSCs cultured *in vitro* with certain characteristics of the disease microenvironment, such as inflammation and hypoxia, in order to alter the biological functions of EVs secreted by MSCs and improve their treatment effects on related diseases (28, 29). Additionally, three-dimensional (3D) cell culture technology has also achieved significant research results in MSCs pretreatment. Compared with two-dimensional culture, 3D culture can be closer to the *in vivo* environment, which helps maintain the pluripotency of MSCs and promotes the secretion of bioactive substances (30, 31).

Although traditional pretreatment strategies, such as inflammatory factor pretreatment, hypoxic culture and 3D culture, simulate certain characteristics of the disease microenvironment to some extent, they do not accurately replicate the ‘personality’ traits of this environment (32). Although the inflammation and hypoxia preconditioning to a certain extent simulates the pathological microenvironmental state of inflammation and hypoxia in some disease lesions, the temporal and spatial nature of the disease microenvironment is ignored. Consequently, the program exhibits a certain degree of lack of specificity, necessitating further refinement and optimization from the perspective of “precision” medicine.

In recent years, research has focused on optimizing the therapeutic effects of MSC-derived exosomes (MSCs-Exos) through the simulation of the disease microenvironment (33). For instance, the pretreatment of mesenchymal stem cells (MSCs) with the extract of tissue homogenate has been demonstrated to enhance the therapeutic effect by targeting the tissue (34). Furthermore, tissue-derived extracellular vesicles (EVs) are regarded as a more promising pretreatment solution, as they can more accurately reflect the characteristics of the disease microenvironment (35, 36). These studies provide a theoretical foundation for developing MSCs-Exos pretreatment strategies that are based on the disease microenvironment.

However, current research on MSC-derived exosome (MSCs-Exos) pretreatment strategies remains in its early stages, with numerous critical issues still to be addressed (37). For instance, how can we more accurately replicate the ‘temporal’ and ‘spatial’ characteristics of the disease microenvironment? Additionally, what engineering transformations can be implemented to further enhance the therapeutic efficacy of MSCs-Exos? Addressing these questions necessitates not only comprehensive foundational research but also interdisciplinary collaboration to facilitate the translation of findings from the laboratory to clinical settings. This review aims to systematically summarize the current advancements in MSCs-Exos pretreatment strategies in relation to the disease microenvironment, investigate the underlying molecular mechanisms and regulatory factors, and evaluate the prospects and challenges associated with clinical translation. By organizing and analyzing existing research, we aspire to offer targeted recommendations for future investigations, advance exosome therapy from basic research to clinical application, and ultimately achieve the objectives of precision medicine.

Finally, taking complex wound repair as an example, we propose the viewpoint of optimizing the intervention scheme and therapeutic effect of MSCs-EVs treatment according to the pathological microenvironment characteristics of the disease to be treated. This hypothesis awaits verification by future experiments to further enhance the safety and effectiveness of mesenchymal stem cell treatment of diseases.

## 2 Mechanism of action of exosomes

### 2.1 Role of exosomes in intercellular communication

Exosomes play a crucial role in the transmission of information between cells. Molecules such as proteins, mRNA, and miRNA carried by exosomes can be internalized by recipient cells, thereby regulating their physiological functions (38). One of the key mechanisms by which extracellular vesicles (EVs) influence intercellular communication is through their ability to modulate the behavior of recipient cells. For instance, EVs derived from stem cells have been shown to promote microglial migration and modulate neuroinflammatory responses. This modulation occurs through specific receptor interactions, such as the activation of purinergic receptors (e.g., P2X4R) on microglial cells, which can enhance their motility and response to injury (39). By targeting these receptors or the signaling pathways they activate, researchers can potentially influence the efficacy of signal transmission via EVs. Furthermore, the composition of EVs can be manipulated to enhance their signaling capabilities. For example, the loading of specific proteins or RNAs into EVs can be optimized to ensure they convey the desired signals to recipient cells. This optimization can be achieved through various methods, including the genetic engineering of donor cells to overexpress certain molecules that are subsequently packaged into EVs (40). Additionally, the use of pharmacological agents to modulate the biogenesis and release of EVs can be explored to enhance their signaling potential. The microenvironment itself significantly impacts the efficacy of EV-mediated communication. Factors such as the presence of inflammatory cytokines, the composition of the extracellular matrix, and overall cellular density can influence the uptake of EVs by recipient cells and the effectiveness of their signal transmission. For instance, in the context of neuroinflammation, the presence of pro-inflammatory cytokines can enhance the uptake of EVs by microglia, thereby amplifying their signaling effects (41). Such mechanisms enable exosomes to selectively deliver specific bioactive molecules tailored to various pathological microenvironments, thereby modulating the physiological functions of recipient cells.

### 2.2 Application of MSC and MSC-Exos in different disease microenvironments

**Figure 2:**
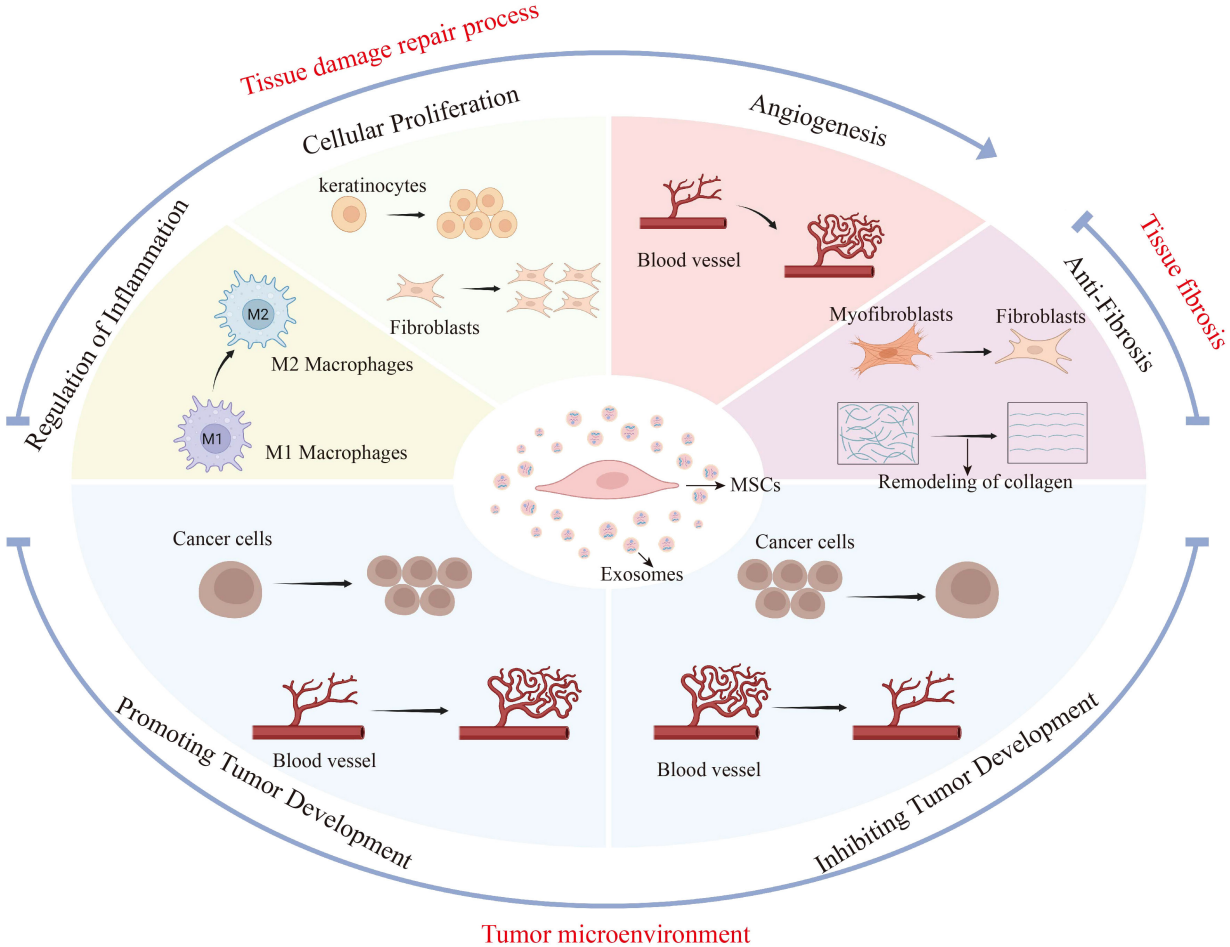
MSC-Exos have different biological effects in different disease microenvironments. This figure illustrates the diverse biological impacts of mesenchymal stem cell-derived exosomes (MSC-Exos) across various disease microenvironments, specifically in tissue repair, fibrosis, and tumor settings. During tissue repair, MSC-Exos regulate inflammation, fostering the shift from M1 to M2 macrophages, and enhance cellular proliferation and angiogenesis to expedite wound healing. In fibrosis, they counteract tissue stiffening by promoting myofibroblast-to-fibroblast transition and collagen remodeling. Within the tumor microenvironment, MSC-Exos may accelerate tumor progression by promoting tumor cell proliferation and angiogenesis. On the other hand, MSC-Exos also inhibited tumor progression by inhibiting tumor cell proliferation and angiogenesis. The figure highlights the influence of the disease microenvironment on the secretory effects of MSC-Exos and the possible promoting or inhibiting effects of MSC-Exos in different pathological states.

The mechanism of exosomes in various pathological microenvironments reflects their high adaptability and specificity (**Figure 2**). Sun et al. has shown that exosomes can selectively transport specific biological agents in accordance with different pathological contexts, thereby exerting distinct therapeutic effects (42).

#### 2.2.1 Tissue repair

MSCs have been successfully applied in multiple tissue repair and disease scenarios. This process is associated with the ability of stem cells to differentiate into fibroblasts, chondrocytes, cardiomyocytes, and endothelial cells, thereby facilitating tissue repair (2, 43–45). For example, in chronic wound healing, MSCs function mainly by enhancing angiogenesis, promoting re-epithelialization, improving granulation formation, and accelerating wound healing (46). As of December 30, 2019, the Clinical Trials Database of the US National Institutes of Health (www.clinicaltrials.gov) has registered 38 clinical trials, mainly investigating the healing potential of MSCs for chronic wounds such as lower extremity ulcers and pressure sores (46). Additionally, in cardiovascular diseases such as myocardial infarction and heart failure, the therapeutic potential of MSCs has been confirmed by most studies. It promotes cardiomyocyte regeneration and angiogenesis through paracrine mechanisms, reverses dysfunction, and restores myocardial function (47). It is noteworthy that notably, adipose-derived mesenchymal stem cells (ADSCs) exhibit differences in their functions in wound treatment. During the process of tissue repair and injury, in the early stage of the same wound healing process, ADSCs can upregulate the expression of collagen I and III in fibroblasts, thereby accelerating collagen synthesis and granulation tissue formation and promoting wound healing (48, 49). However, after the epithelialization of the wound is completed, the paracrine effect of ADSCs in the later stage of healing manifests as downregulation of collagen synthesis and antagonism of fibrosis or scar formation (26, 50). These findings reveal the complex roles of mesenchymal stem cells under different disease and transplantation conditions.

Similarly, the use of MSC-Exos for tissue repair has also been verified. As an alternative cell-free therapeutic approach, MSC-Exos can transfer molecules such as mRNAs, miRNAs, proteins, and lipids to the local microenvironment and recipient cells in either a direct or indirect manner (51, 52). In so doing, they simulate the biological activity of mesenchymal stem cells, promote the activation of endogenous repair mechanisms, and accelerate tissue regeneration and functional restoration (53). For example, in wound repair, MSC-Exos enhance the biological characteristics of keratinocytes, fibroblasts, and endothelial cells (54). In myocardial repair, Exos have demonstrated therapeutic effects in preclinical models of ischemic heart disease (55).

Overall, MSCs and MSC-Exos function in tissue injury repair mainly by promoting angiogenesis, re-epithelialization, and granulation formation, as well as regulating immune and inflammatory responses and inhibiting apoptosis. The main repair mechanism of MSCs is the paracrine secretion of growth factors and cytokines. MSC-Exos, as bioactive vesicles secreted by MSCs, have potential as a new cell-free treatment.

#### 2.2.2 Tissue fibrosis

MSCs have the potential to treat fibrotic diseases such as pulmonary fibrosis and liver fibrosis by regulating inflammatory responses, downregulating the expression of fibrosis-related genes, exerting anti-inflammatory and antioxidant effects, and promoting tissue repair (56–59). For example, ADSCs can reverse bleomycin-induced pulmonary fibrosis, as shown by decreased neutrophil infiltration, repair of damaged tissues, reduced collagen deposition, and inhibition of the NF-κB signaling pathway (60).

MSC-Exos exhibit similar therapeutic efficacy in fibrotic diseases (61). In a carbon tetrachloride (CCl4)-induced fibrotic liver model, HUC- MSC-Exos mitigated liver fibrosis by reducing the number of fibrous capsules, softening the texture, and decreasing inflammation and collagen deposition (62). This outcome is also thought to be associated with the TGF-β1/Smad2 signaling pathway (63, 64). Additionally, some studies have shown that MSC-Exos can alleviate fibrosis by reprogramming macrophage polarization by delivering miR-148a to macrophages and targeting the KLF6/STAT3 pathway (65). Moreover, MSC-Exos exert an antirenal fibrotic effect via miRNA-122a (66).

#### 2.2.3 Tumor microenvironment

The use of MSCs and their Exos has led to certain advances in the treatment of tumors, yet there is also controversy (67–71). In terms of promoting tumors, bone marrow mesenchymal stem cells (BMSCs) can fuse with tumor cells through direct or indirect interactions and migrate to the tumor site, exhibiting a stronger proangiogenic effect *in vivo* and *in vitro* and promoting tumor growth (72). Another study revealed that the growth factors and cytokines secreted by dental pulp MSCs increase the expression of Ki-67 in oral cancer cells, suggesting the possibility of promoting cancer development (73). However, MSCs also have antitumor effects. After entering the body of cancer patients, MSCs can effectively reduce the rate of cancer cell expansion by regulating immune components in the tumor environment (74). Research has also indicated that MSCs and HUC-MSCs can inhibit tumor growth by regulating the phenotype of inflammatory cells around tumors (75). Another study revealed that the secretome of HUC-MSCs exerts an anticancer effect by inducing the apoptosis of MCF-7 tumor cells (76). In addition, the antitumor activity of MSCs, which inhibits tumor angiogenesis, has also been proven (77).

Furthermore, MSC-Exos are capable of transmitting signal molecules to regulate tumor cell proliferation, angiogenesis, and metastasis (78), and they can also promote tumor resistance (79, 80). Additionally, MSC-Exos can also act as carriers for targeted tumor therapy (81–84). For example, BMSC-Exos loaded with doxorubicin target the treatment of osteosarcoma through the SDF1–CXCR4 axis (85).

As mentioned, MSCs and MSC-Exos can influence tumor progression through various pathways, exhibiting both pro-tumor and anti-tumor effects. On one hand, they promote tumor growth, angiogenesis, invasiveness, and drug resistance; on the other hand, they can inhibit tumors by modulating immune responses and intercellular signaling (86). This dual effect, while complicating tumor treatment, allows for personalized therapeutic strategies to be developed based on the patient’s tumor type and pathological characteristics through the optimization of precise regulation of MSCs and MSC-Exos, providing additional support to existing treatment regimens (87). Although numerous studies have reported on the relevant effects of MSC-Exos, the biological transformations of MSC-Exos may vary significantly in different environments, and they contain various substances with complex biological activities, such as miRNAs, which show significant differences across different Exos Furthermore, Exos miRNAs participate in vast effect networks and may exert different or similar biological effects through various pathways. Therefore, our comprehensive understanding of their mechanisms remains very limited, and the controllability of their effects has yet to be fully validated. In the future, it is essential to explore more innovative research methods to further unveil their potential.

Further contradictions arise from the differences in experimental design across studies.Exos can be isolated from various tissues, including bone marrow, adipose tissue, umbilical cord, dental pulp, and placenta (88, 89). There are certain differences in the proteins, RNAs, and other components contained in MSC-Exos from different sources or with different pre-treatments (90). Any inconsistency at any stage may lead to varying effects on tumors. Moreover, different tumor types respond differently to the same MSC-Exos;For instance, exosomes derived from adipose-derived stem cells (ADSC-Exos) can enhance the anti-tumor effect of natural killer T (NKT) cells *in vivo*, which in turn contributes to the inhibition of hepatocellular carcinoma (HCC) (91) and they can also activate the Wnt signaling pathway to promote the migration of the MCF-7 human breast cancer cell line (92). The source, injection dosage, timing of administration, and tumor type of MSC-Exos collectively influence their biological effects in tumors (78). Additionally, the lack of standardized techniques for the extraction, purification, and storage of MSC-Exos further exacerbates the inconsistency of results (81). Therefore, there is a pressing need for a more comprehensive exploration of the mechanisms by which MSCs and MSC-Exos exert effects in tumors. It is essential to systematically elucidate the mechanisms of MSCs/MSC-Exos and establish standardized systems for preparation, storage, and functional assessment to enhance the reproducibility of research and accurately define their potential and risks in tumor therapy.

## 3 Temporal and spatial attributes of disease microenvironments

### 3.1 Complexity of the disease microenvironment

The complexity of the disease microenvironment is characterized by a diverse array of cell types that play crucial roles in disease progression (93, 94). We propose that, aside from laboratory conditions and systemic drug use, the varying effects exhibited by MSCs may stem from distinct pathological microenvironments associated with different organs and tissues, or varying stages of the same disease. These microenvironments possess unique features capable of inducing significant differences in the paracrine effects of MSCs. For instance, in the context of chronic wound healing, the micro-environment of wounds exhibits significant differences during the inflammatory, proliferation, and remodeling phases (95). During the inflammatory phase, the micro-loop is abundant in inflammatory cells and cytokines, such as TNF-α and IL-1β, which facilitate the removal of pathogens and necrotic tissue (96). In the proliferation phase, the microenvironment contains a wealth of growth factors and cells, alongside the extracellular matrix, which promotes cell proliferation and tissue repair (97). Finally, during the remodeling phase, the micro-ring is characterized by collagen deposition and tissue maturation (98).

Our review of the literature indicates that in chronic infections, inflammations, and cancers, the tissue microenvironment plays a crucial role in governing the behavior of local immune cells (99). Fibroblasts residing in diseased tissues are essential in regulating the activation or suppression of immune responses (100). These resident fibroblasts may originate from the circulation (including bone marrow-derived precursors and bone marrow-derived mesenchymal stem cells), local diseased tissues, or adjacent tissue cells such as adipocytes, adipose-derived mesenchymal stem cells, and pericytes (99).

### 3.2 Fibroblast heterogeneity and disease microenvironment

The fibroblast plays a crucial role in shaping the tissue microenvironment, and fibroblasts from different sources play distinct roles in disease progression (100–102). However, its heterogeneity is closely linked not only to the tissue source but also to the specific characteristics of its anatomical location. A study identified 88 coding genes that were expressed at higher levels in lung fibroblasts relative to fibroblasts from other tissues, highlighting the unique transcriptional landscape associated with pulmonary fibroblast (103). Even fibroblasts from the same anatomical site can exhibit a range of phenotypes, influenced by local microenvironmental factor (104). For example, fibroblasts in the dermis can be categorized into distinct subpopulations, such as papillary and reticular fibroblasts, each contributing differently to the extracellular matrix (ECM) and tissue homeostasis (105). The papillary fibroblasts are known for their role in supporting epidermal maintenance, while reticular fibroblasts are more involved in the structural integrity of the dermis. Furthermore, the heterogeneity of fibroblast populations is closely associated with the overall health of the human body (106). In healthy tissues, fibroblasts maintain a balance that supports tissue homeostasis. However, in diseased states, the signals from damaged tissues, inflammatory cells, and the ECM can lead to the activation of fibroblasts and their subsequent differentiation into various subpopulations, each with distinct roles in the pathophysiology of the disease (107, 108).

Fibroblasts not only possess distinct tissue characteristics but also display significant temporal and spatial properties, enabling them to play rich and complex roles in the evolution and progression of diseases (106). Sole-Boldo et al. identified four populations of dermal fibroblasts through RNA-seq analysis, each with different functional transcriptome features and spatial distribution characteristics (109). Moreover, subbasal papillary fibroblasts have active Wnt signals, while reticular dermal fibroblasts highly express genes related to the ECM and immune signal transmission (106). Therefore, some researchers have summarized four key variables that determine the extensive heterogeneity of fibroblasts: tissue state, regional characteristics, microenvironment, and cell state (106). It is precisely the existence of this disparity that leads to the fact that even for the same disease, its pathological microenvironment will exhibit unique characteristics due to differences in disease location and disease stage.

For instance, in cases of tissue repair, organ fibrosis, and solid tumors, these diseases are closely associated with fibroblasts and each possesses a distinct pathological microenvironment (99). Particularly, carcinoma-associated fibroblasts (CAFs) have a strong connection between the heterogeneity of their subpopulations and the tumor microenvironment they reside in (110). This heterogeneity not only offers a new perspective for our understanding of tumor occurrence and development but also implies that there are tremendous opportunities and challenges on the path of future tumor precision medicine and individualized treatment.

Similarly, in skin wound repair, the external and internal environments of the skin wound are relatively independent yet interact with each other and are invariably closely associated with the dynamic pathophysiological changes throughout the repair process (111). Considering the alterations in the pathological microenvironment, it is believed that in the early stage of treatment after injury, greater emphasis might be placed on controlling inflammation and related cell proliferation. In contrast (112, 113), in the later stage, more attention may be required to focus on tissue repair and functional restoration. Generally speaking, the pathological microenvironment is a multifaceted entity that demands in-depth research to unveil its complexity and formulate effective treatment strategies.

In summary, fibroblasts, which are closely related to the disease microenvironment, exhibit extensive heterogeneity due to differences in tissue status, regional characteristics, cell status, and the microenvironment they inhabit. This heterogeneity can lead to variations in the pathological microenvironment of the same disease, depending on the organ or tissue involved and the stage of disease progression. Therefore, we speculate that the “unique” pathological microenvironment characteristics of diseases closely related to fibroblasts, such as wounds, cutaneous scars, organ fibrosis, and solid tumors, possess “tissue/site” attributes and “disease stage/progression” attributes. Transplanted MSCs can secrete intervention or therapeutic secretomes in response to the “unique” pathological microenvironments of the same disease at different sites (e.g., solid tumors at different locations) or even the same disease at the same site but at different stages of development (open wounds versus scars, or different stages of tumor progression). This may be one of the important reasons why MSCs have been proven to be effective in treating a variety of human diseases (2). Similarly, in addition to the above-mentioned laboratory factors, it may be that researchers have ‘neglected’ the ‘temporal’ and ‘spatial’ attributes of the disease microenvironment when studying the paracrine effects of MSCs, or that it is precisely because of the differences in ‘time’ and ‘space’ of the pathological microenvironment of the same disease or similar diseases that different researchers’ research results are inconsistent (114).

## 4 The influence of pathological microenvironment on the secretion of MSC-Exos

### 4.1 Effect of pathological microenvironment on MSCs

Inflammation and hypoxia have been utilized as pretreatment approaches for MSCs cultured *in vitro* to boost the therapeutic effectiveness of corresponding MSCs-Exos for diverse diseases (28, 29). For example, the research of Yu et al. emphasizes that inflammatory cytokines such as interleukin-1β (IL-1β) and interferon-γ (IFN-γ) can impact the immune characteristics of human umbilical cord blood-derived MSCs (115). Antebi et al. reported that MSCs under hypoxic conditions possess enhanced therapeutic attributes (116, 117). Under short-term hypoxic circumstances, MSCs show accelerated cell proliferation, upregulated expression of VEGF, inhibited pro-inflammatory cytokine interleukin-8(IL-8), increased anti-inflammatory cytokine IL-1RA, and downregulated expression of apoptotic genes BCL-2 and CASP3. On the contrary, under long-term hypoxic culture conditions, the proliferation rate of MSCs is significantly decelerated and the yield is lower. Continuous pathological stimulation may affect the long-term survival and biological functions of MSCs, leading to a reduction in therapeutic effects (118). These pretreatment strategies based on the characteristics of the pathological microenvironment have begun to emerge as an important means to enhance the biological effects of MSCs.

Furthermore, there are certain highly expressed factors in the pathological microenvironment, such as fibronectin and laminin, which might facilitate the adhesion and localization of MSCs (119). In tissue repair following injury, inflammation recruits MSCs to the damaged tissue. Simultaneously, the pro-inflammatory factors generated by MSCs play a crucial role in tissue repair (120). Yuan et al. also discussed that the changes in the local microenvironment of the liver are of great significance for the homing of MSCs and can enhance the efficacy of liver disease treatment (121). This indicates that inflammatory factors are involved in the recruitment of MSCs to the injured site and further supports the notion that the pathological microenvironment plays an important role in influencing the homing of MSCs.

### 4.2 Effect of pathological microenvironment on MSC-Exos secretion

The microenvironment in which MSCs reside has a profound influence on their paracrine signaling capacity and therapeutic efficacy (13). Research indicates that changes in the microenvironment, such as high levels of extracellular adenosine, can modify the secretome of MSCs and influence their interaction with other cell types (122). Recent studies have highlighted the significance of considering the microenvironment when investigating the potential therapeutic applications of MSC-Exos (123).

#### 4.2.1 The pathological microenvironment alters the biological function of MSC-Exos

Specific factors within the pathological microenvironment can modify the composition of microRNAs (miRNAs) and proteins in extracellular vesicles of MSCs, thereby influencing the gene expression and function of recipient cells. For instance, Kim et al. investigated the anti-inflammatory effect and mechanism of extracellular vesicles secreted by MSCs pretreated with interleukin-1β (MSC-IL-Exos) in osteoarthritis SW982 cells. Compared to extracellular vesicles from MSCs without IL-1β treatment, MSC-IL-Exos not only inhibited the expression of pro-inflammatory cytokines but also enhanced the expression of anti-inflammatory factors. The study confirmed that MSC-IL-Exos mediate anti-inflammatory effects through miRNAs such as miR-147b, involving the inhibition of the nuclear factor kappa-B (NF-κB) pathway (124). The study by Cheng et al. also corroborated this. The EVs obtained by pretreating MSCs with IL-1β by their team enhanced the therapeutic effect of MSC-Exos in an *in vitro* lipopolysaccharide (LPS)-induced sepsis model. This indicates that pretreatment may enhance certain characteristics of Exos, thus playing a more effective role in the inflammatory response (125). In Han’s review, it is also mentioned that MSCs pretreated with interferon-γ (IFNγ), tumor necrosis factor-α (TNFα), or IL-1 may exert their immune effects by altering the biological characteristics of the extracellular vesicles they secrete (126).

#### 4.2.2 The pathological microenvironment regulates the secretion level of MSC-Exos

Certain factors within the pathological microenvironment can enhance the biological characteristics of Exos by elevating the secretion level of MSC-Exos. These pathological elements interact with the surface receptors of MSCs, activating signal transduction pathways related to the release of Exos and thereby promoting the secretion of Exos. Zhang et al. noted that under hypoxic conditions, hypoxia-inducible factor (HIF-1α) can mediate a significant upregulation in the expression of nSMase2 (127). And nSMase is a crucial regulator of Exos secretion, facilitating an increase in Exos secretion. Through increasing the secretion quantity of MSC-Exos, changes in the pathological microenvironment can not only raise the therapeutic concentration of Exos in the disease microenvironment but also endow Exos with more abundant biological characteristics, such as specificity. For instance, Nakao et al. discovered that pretreatment of human gingiva-derived mesenchymal stem cells (GMSCs) with TNF-α can not only increase the number of secreted Exos but also enhance the expression of CD73 in Exos, thereby inducing anti-inflammatory M2 macrophage polarization and strengthening the anti-inflammatory effect of Exos (128).

However, in the pathological microenvironment of tumors, the increased expression of MSC-Exos has a detrimental effect. The acidic condition in the tumor microenvironment may alter the secretion of Exos. Logozzi et al. verified and characterized the classic surface markers of Exos through Western Blotting and confirmed that the Exos released by tumor cells cultured in an acidic environment are significantly increased. Promoting the release of Exos means promoting and maintaining tumor progression (129). Wu et al. reported that the stiffness of the ECM is also one of the factors for changes in the pathological microenvironment. The rigid ECM can promote the release of Exos by tumor cells, which can activate the Notch signaling pathway and thereby promote tumor growth (130).

### 4.3 Summary

The complexity of the pathological microenvironment exerts multifaceted influences on MSCs and MSC-Exos. Thorough research is required to uncover its influence mechanism and offer new strategies for the precise treatment of diseases. The pathological microenvironment characteristic factors employed in existing studies as pretreatment methods for MSCs may not fully mimic the actual pathological environment of diseases. Hence, it is essential to fully consider the heterogeneity of disease-related fibroblasts and study the interactions between fibroblasts and MSCs as well as MSC-Exos in different disease microenvironments. We are hopeful of providing new strategies and methods for the precise treatment of diseases. In the subsequent content, we will focus on three types of diseases with inflammatory pathological features, namely tissue injury repair, tissue fibrosis, and tumor diseases. We will analyze the treatment status of MSCs and their Exos and explore the therapeutic effects of MSC-Exos obtained after inflammatory pretreatment of MSCs in these three types of diseases.

## 5 Traditional preconditioning strategies and their limitations and challenges

### 5.1 Effect of inflammation pretreatment

Inflammatory factor pretreatment of MSCs will cause changes in the contents of their EVs, thereby exerting regulatory effects on cell communication and physiological processes. For example, TNF-α pretreatment can strengthen the osteogenic differentiation capacity of MSCs (131). Research has shown that exposing MSCs to inflammatory stimuli can lead to significant changes in their gene expression profiles, enhancing their ability to modulate immune responses and promote healing. Szűcs et al. have indicated that preconditioning MSCs with pro-inflammatory cytokines like TNF-α can elevate the expression of various interleukins, chemokines, and growth factors that are crucial for wound healing and tissue regeneration (132). This pre-activation can potentially lead to stronger wound healing responses and improved outcomes in conditions such as spinal cord injury (SCI) and other inflammatory diseases. Similarly, pretreatment with hypoxia and inflammatory factors (IL-1β, TNF-α, IFN-γ) can boost immunomodulatory effects without undermining biological properties (133).

However, owing to the complexity of the disease microenvironment, the therapeutic effects of MSCs following inflammatory pretreatment still vary in different diseases. One significant concern is the risk of inducing a pro-inflammatory phenotype in MSCs, which may lead to adverse effects rather than the desired therapeutic outcomes. The inflammatory environment can sometimes push MSCs towards a more inflammatory state, which could exacerbate tissue damage instead of promoting healing (132). Another limitation is the variability in response to inflammatory preconditioning among different MSC populations. Factors such as the source of MSCs (e.g., bone marrow, adipose tissue, umbilical cord), the specific inflammatory stimuli used, and the duration of exposure can all influence the outcome. This variability can complicate the standardization of preconditioning protocols, making it difficult to predict the behavior of MSCs in clinical settings (132, 134). From the perspective of precision medicine, we need to further refine and perfect pretreatment methods such as inflammation to increase the specificity of MSCs-Exos in treating different diseases and explore the mechanism of action of MSCs-Exos in diverse pathological microenvironments.

### 5.2 Effect of hypoxic pretreatment

Hypoxic preconditioning activates HIF-1α in MSCs by simulating the hypoxic microenvironment present *in vivo*, thereby enhancing the secretion of factors such as VEGF and HIF-1α. These factors play crucial roles in ischemic tissues, facilitating vascular neovascularization and tissue repair (135). Hypoxic preconditioning improves the therapeutic efficacy of MSCs-Exos. Research has shown that hypoxic preconditioning of human umbilical vein endothelial cells (HUVECs) enhances the angiogenic capacity of MSCs by stimulating the secretion of exosomes that promote MSC-mediated tube formation and effective nerve tissue repair in a rat model of spinal cord injury (136). Furthermore, Wang et al. have demonstrated that hypoxic preconditioning of BMSCs enhances the therapeutic efficacy of their derived exosomes (Hypo-Exos) in promoting facial nerve repair and regeneration (137).

Although hypoxic preconditioning has demonstrated significant efficacy in laboratory studies, it continues to face numerous challenges in clinical translation. First, variations in hypoxia concentrations and treatment durations significantly impact the biological properties of MSCs. The long-term effects of hypoxic preconditioning on stem cell behavior and function remain to be fully elucidated. While short-term exposure to hypoxia can enhance stem cell properties, prolonged hypoxic conditions may lead to unintended consequences, such as altered differentiation pathways or increased senescence, which could compromise the regenerative potential of the cells. Second, discrepancies in hypoxic conditions (e.g., oxygen concentration and treatment duration) employed across different laboratories have resulted in inconsistent findings, thereby affecting the feasibility of clinical translation (138–140).

### 5.3 Challenges

The existing pretreatment methods often overlook the unique characteristics of the disease microenvironment, which restricts their potential for clinical application (141). For instance, the addition of osteogenic factors can enhance the osteogenic differentiation of MSCs, while other factors may promote adipogenic or chondrogenic differentiation (142). the microenvironmental features of different diseases and individual patients can vary significantly, making it crucial to develop individualized pretreatment protocols tailored to specific circumstances in order to enhance therapeutic efficacy. Furthermore, pretreatment programs must achieve a higher level of precision to fulfill the requirements for clinical applications. For example, by simulating specific conditions present in the disease microenvironment (such as inflammation and hypoxia), preconditioning mesenchymal stem cells (MSCs) can enhance the functionality of their secreted exosomes, thereby improving therapeutic outcomes (28, 29).

## 6 Preconditioning strategies by mimicking disease microenvironments

### 6.1 Pretreatment of homogenates based on disease tissue

The use of tissue homogenate supernatant to simulate a specific microenvironment is a common pretreatment method for MSCs. Xue et al. utilized mouse liver tissue homogenate supernatant to simulate the liver microenvironment and successfully induced HUC-MSCs to differentiate into liver-like cells with hepatocyte phenotype and function (143). Yang et al. also employed mouse brain tissue homogenate supernatant to simulate the brain tissue microenvironment, which can upregulate the secretion of brain-derived neurotrophic factor (BDNF) by progesterone-induced HUC-MSCs (144).

Additionally, Hao et al. used wound tissue homogenate supernatant in the inflammatory stage to simulate the wound microenvironment for pretreating MSCs. *In vitro* experiments demonstrated better effects on promoting fibroblast proliferation and migration than traditional culture conditions (145). At the same time, Wang et al. adopted wound tissue homogenate combined with inflammatory factors to pretreat MSCs (146). The results showed that MSC-Exs obtained by this approach are superior to those under traditional culture conditions in regulating wound inflammation and promoting repair. The above studies have confirmed our previous supposition that wound tissue homogenate simulates the pathological microenvironmental factors in the inflammatory stage of the wound. Moreover, the tissue homogenate combined with inflammation group is superior to the simple tissue homogenate group. Although using wound tissue homogenate supernatant to simulate the wound microenvironment can theoretically simulate the disease microenvironment to a certain extent, the disease microenvironment has “spatio-temporal” characteristics. Designing pretreatment schemes according to the spatio-temporal characteristics of wounds can become a future research direction.

### 6.2 Preprocessing based on disease tissue-derived EVs

Recent investigations have revealed that EVs originating from tissues hold extensive application prospects in disease research. For example, Brenna et al. characterized EVs within the brain tissue of mice under physiological states and following transient cerebral ischemia (147). Under steady-state conditions, microglia serve as the predominant source of EVs. In contrast, after cerebral ischemia, the major EVs are derived from astrocytes. Furthermore, the EVs released by astrocytes exhibit a further increase post-stroke. This research indicates that EVs sourced from brain ischemic tissue can reflect the pathological alterations in the brain tissue spatially following cerebral ischemia. These tissue-derived EVs are capable of reflecting tissue-specific pathological changes and participating in multiple physiological and pathological processes, such as angiogenesis and immune regulation. In comparison to EVs isolated from body fluids, EVs directly isolated from tissues possess advantages such as tissue specificity and accurately reflecting the tissue microenvironment (35, 36). In addition, currently, tumor tissue-derived EVs have been applied in multiple aspects, including tumor diagnosis, disease staging, progression assessment, and evaluation of treatment efficacy (148).

In conclusion, the relatively simple and controllable composition of tissue-derived EVs as key messengers for intercellular information exchange, the ability of their source cells to more accurately respond to the pathological state of disease by growing in the three-dimensional microenvironment of diseased tissues, and their property of analyzing the spatial and temporal heterogeneity of the tissue microenvironment, have collectively led to the establishment of preliminary guidelines for laboratory quality control and enhanced prospects for clinical translation (148). It is therefore anticipated that EVs derived from diseased tissue will become a more promising model for representing the pathological microenvironment of diseased tissues. To further substantiate our hypothesis, we propose that subsequent studies could employ disease tissue-derived EVs to mimic the *in vitro* microenvironment of the disease. Consequently, the EVs that can be obtained after intervening with the MSCs would be more specific for the treatment of the disease. This pre-treatment strategy of the MSCs is worthy of further investigation.

### 6.3 Challenges

Despite the significant potential demonstrated by the use of disease tissue-derived homogenates and Exos to simulate microenvironments for preconditioning MSCs, their reproducibility and standardization remain major bottlenecks for clinical implementation. Firstly, any research involving the procurement of human tissues must undergo rigorous ethical review; researchers are required to provide a detailed research plan outlining how tissue samples will be obtained and utilized, ensuring the privacy of donors is protected (149). This process often takes a considerable amount of time. In microenvironment simulations, tissue homogenates and EVs derived from healthy donors may serve as effective controls. However, it is sometimes challenging to collect healthy tissue or it may not meet ethical standards. Furthermore, when collecting pathological tissue, strict inclusion and exclusion criteria should be established in advance. In reality, the acquisition of high-quality disease tissue samples is very limited, and variations among different donors may affect the quality of tissue homogenates or EVs. Tissue homogenates themselves exhibit high complexity, as they contain a vast array of highly diverse information, such as DNA, RNA, proteins, lipids, and polar metabolites, with significant variations in their content and properties across different tissues and processing conditions (150). Although various preparation schemes have been reported, the method for preparing tissue homogenates still requires continuous optimization. The variability in the methods for extracting EVs from tissue sources is influenced by multiple factors, including the type of tissue used, the effects of mechanical and enzymatic treatments on cells and EVs, the incubation time for tissue to release EVs, the number of separable Exos subpopulations, and the type of EVs isolation method employed (151, 152). Furthermore, different storage solvents, temperatures, and storage durations also impact the quality of EVs (153, 154). From 2014 to 2023, the evolution of the Minimal Information for Extracellular Vesicles (MISEV) guidelines reflects the ongoing efforts in this field to enhance research reproducibility and standardization (18).

In summary, advancing the preparation of tissue homogenates and tissue-derived EVs to clinical-grade Good Manufacturing Practice (GMP) production still faces multiple challenges. Donor heterogeneity and insufficient traceability of pathological tissue collection lead to significant inter-batch variability, affecting the consistency and reliability of the extracts. Furthermore, the lack of uniformity in ethical review processes and the absence of cross-center standards limit its widespread clinical application. Additionally, there is currently a lack of unified GMP-level production specifications, and critical quality attributes have not been clearly defined. To address these challenges, it is recommended to establish a rigorous donor screening and traceability system, while also formulating quality control standards and guidelines that comply with GMP requirements. These measures will help improve the quality, safety, and feasibility of its clinical translation.

## 7 Molecular mechanisms and regulatory factors

### 7.1 Role and regulation of miRNA

miRNAs in exosome form are pivotal molecules that regulate the function of receptor cells. miRNAs are a class of small non-coding RNAs that are able to regulate gene expression by binding to mRNAs, thereby either inhibiting their translation or promoting their degradation (155). Studies have demonstrated that miRNAs can be selectively absorbed by exosome and delivered to recipient cells to regulate their physiological activities (156). These non-coding molecules have been demonstrated to play pivotal roles in various aspects of cellular communication, including cell proliferation, differentiation, apoptosis, and inflammatory responses (156).

At present, EVs or exosome-derived miRNAs have become a focal point in research endeavors aimed at the diagnosis and treatment of a wide range of diseases, including tumors (157), trauma (158), and metabolic diseases (159). In the study of MSCs-Exos, researchers identified a variety of key microRNAs (miRNAs) that regulate therapeutic effects through high-throughput sequencing and bioinformatics analysis. These identified miRNAs play important roles in promoting tissue repair, suppressing inflammation, and regulating immune responses (160, 161). For instance, miR-126, which is highly expressed in MSC-Exos, has been shown to promote bone fracture healing by SPRED1/Ras/Erk signaling pathway (162).Additionally, the anti-inflammatory effects of miR-146a in MSCs-Exos were demonstrated by its capacity to attenuate inflammatory responses by targeting the NF-κB signaling pathway (163).Furthermore, miR-27b-3p, identified in MSC-Exos, has been shown to inhibit fibrosis by downregulating YAP/LOXL2 pathway. This indicates the potential of miR-27b-3p in treating fibrotic diseases such as liver fibrosis (164).

In conclusion, The loading of these key miRNAs into MSC-Exos to enhance efficacy has emerged as a novel research direction (159, 165). These studies lay the theoretical foundation for the engineering of modified exosome-based therapies. By loading specific microRNAs (miRNAs), the therapeutic efficacy of these vesicles can be enhanced, thus rendering them more widely applicable in a variety of diseases.

### 7.2 Application of engineered exosomes

By loading key miRNAs and other molecules to construct engineered exosome, specific therapeutic effects can be enhanced. Utilizing genetic engineering and nanotechnology, researchers have successfully augmented therapeutic outcomes by loading specific miRNAs or proteins into these vesicles, enabling the targeted release of these molecules upon reaching the intended cells, thereby improving treatment efficacy. For example, in the treatment of chronic wounds associated with diabetes, Yang et al. confirmed that engineered exosomes containing miR-31-5P significantly promoted angiogenesis, thereby enhancing blood vessel formation and facilitating the healing of diabetic wounds (166). In tumor therapy, engineered exosomes loaded with miR-317b-5p have been shown to effectively target and alter the bioactivities of tumor cells, leading to reduced tumor growth and improved survival rates in animal models (167).

In recent years, researchers have engineered exosomes using various methods, including electroporation, heat shock, saponin permeabilization, and cholesterol modification; however, these methods often result in low loading efficiency (168). Additionally, transfection reagents such as Exo-Fect have shown high transfection efficiency for miRNA, but Exo-Fect can interfere with the membrane structure of Exos, promoting Exos aggregation and altering their membrane barrier properties and surface charge, which may affect the biological functions of Exos and their subsequent applications. Surface modification of Exos enables targeted delivery. Researchers achieved targeted delivery to ischemic brain injury regions by coupling the cyclo(Arg-Gly-Asp-D-Tyr-Lys) peptide [c(RGDyK)] to the surface of Exos (169). Furthermore, by inserting cationic motifs on the surface of Exos, their transport rate and retention time were enhanced (170). Similarly, positively charged Exos have been utilized in gene therapy for osteoarthritis, improving their delivery efficiency in cartilage by reversing the charge (171). RNA nanotechnology-modified galactosamine (GalNAc)-decorated Exos have served as an effective means for targeted delivery to liver cancer, as GalNAc binds to the overexpressed asialoglycoprotein receptor (ASGP-R) on liver cancer cells, endowing the Exos with targeting capability (172). These methods can effectively enhance the loading efficiency of miRNA into Exos and their functional delivery capabilities. However, optimizing these methods to balance efficacy, stability, and safety remains a significant challenge. Currently, the relevant findings are primarily at the preclinical stage, with further extensive clinical applications yet to be developed.

In summary, engineered exosomes loaded with key effector miRNAs hold significant promise for a range of therapeutic applications, including targeted drug delivery, cancer treatment, and the investigation of inflammatory processes. Their capacity to efficiently deliver miRNAs and selectively target specific pathways paves the way for novel therapeutic interventions. Consequently, engineered exosomes loaded with miRNAs are likely to play an increasingly vital role in the development of innovative therapies for various diseases.

## 8 Conclusion and future perspectives

This review reviews the biological effects of MSCs and MSC-Exos in the treatment of three different pathophysiological states: tissue repair, fibrosis, and tumor, and analyzes the impact of the disease microenvironment on MSCs function and paracrine signaling. This highlights the importance of individual differences in the disease microenvironment and dynamic changes in disease development for precision medicine. The in-depth understanding of the disease microenvironment makes us more demanding for precise treatment. Based on this, we have reviewed the research status of MSC-Exos treatment and proposed the “extracellular vesicle hypothesis of mesenchymal stem cell microenvironment” (173). MSC-Exos with enhanced therapeutic effects can be obtained by simulating the disease microenvironment of MSCs pretreatment. Finally, the engineered exosomes loaded with mirnas with key effects were prepared to improve the therapeutic effect. This strategy not only improves the targeting and effectiveness of MSC-Exos, but also provides a new idea for precision medicine.

Looking forward to the future, we look forward to more about MSC-Exos treatment research, to confirm or refute this review. Through continuous exploration and optimization, the safety and effectiveness of mesenchymal stem cells in the treatment of diseases can be improved, and the quality of life of patients can be improved.
